# Adequate Intake of Dietary Fiber May Relieve the Detrimental Impact of Blood Lead on Dyslipidemia among US Adults: A Study of Data from the National Health and Nutrition Examination Survey Database

**DOI:** 10.3390/nu15204434

**Published:** 2023-10-19

**Authors:** Bin Li, Fengzhan Zhang, Haoqi Jiang, Chen Wang, Qihong Zhao, Wanshui Yang, Anla Hu

**Affiliations:** Department of Nutrition and Food Hygiene, Center for Big Data and Population Health of IHM, School of Public Health, Anhui Medical University, 81 Meishan Road, Hefei 230032, China; lb19990920@163.com (B.L.); zfz2286880392@163.com (F.Z.); jhq0716@163.com (H.J.); unciawang@126.com (C.W.); qihong@ahmu.edu.cn (Q.Z.); wanshuiyang@gmail.com (W.Y.)

**Keywords:** lead, dyslipidemia, dietary fiber, National Health and Nutrition Examination Survey

## Abstract

Lead (Pb) exposure is a well-established risk factor for dyslipidemia, and people are exposed to it in multiple ways daily. Dietary fiber is presumed to improve lipid metabolism disorders, but it is still unknown whether it can relieve the detrimental impact of Pb on dyslipidemia. We used publicly accessible data from the 2011–2016 cycles of the National Health and Nutrition Examination Survey (NHANES). A total of 2128 US adults were enrolled for the subsequent analysis. Heavy metal concentrations in blood were measured using inductively coupled plasma mass spectrometry (ICP-MS). A weighted logistic regression was conducted to calculate odds ratios (ORs) and 95% confidence intervals (CIs). The dose–response relationship between blood heavy metals and dyslipidemia was explored using a weighted restricted cubic spline (RCS) analysis. After fully adjusting for potential confounding factors (age, gender, race, education level, ratio of family income to poverty, marital status, body mass index, physical activity, waist circumference, smoke, alcohol drinking and history of metabolic syndrome, hypertension, and diabetes), a positive association between blood Pb levels and dyslipidemia risk was revealed (OR = 1.20, 95% CI: 1.03–1.40). Dietary fiber intake may significantly modify the association between blood Pb levels and dyslipidemia (*p*-interaction = 0.049), with a stronger association (OR = 1.26, 95% CI: 1.05–1.52) being revealed in individuals with an inadequate intake of dietary fiber (<14 g/1000 kcal/day), but a null association (OR = 1.01, 95% CI: 0.72–1.42) being observed in those with an adequate intake of dietary fiber (≥14 g/1000 kcal/day). Moreover, the weighted RCS analysis showed that compared with the average blood Pb exposure level (4.24 µg/dL), a lower blood Pb exposure level (3.08 µg/dL) may contribute to the risk of dyslipidemia in the group with an inadequate dietary fiber intake. Our findings suggest that Pb exposure in blood may be a risk factor for dyslipidemia. However, an adequate dietary fiber intake may offset the risk of dyslipidemia caused by blood Pb exposure. Since avoiding Pb exposure in daily life is difficult, increasing dietary fiber intake in the future might be a promising approach to alleviate dyslipidemia caused by Pb exposure.

## 1. Introduction

Dyslipidemia, the abnormal metabolism of lipids, is a leading risk and preventable factor for metabolic syndrome, insulin resistance, and cardiovascular disease [1,2,3]. The diagnostic indicators of dyslipidemia involve triglyceride (TG), total cholesterol (TC), LDL-cholesterol (LDL-C), and HDL-cholesterol (HDL-C), and the most common type of dyslipidemia is hypercholesterolemia [4]. The World Health Organization estimated that almost 39% of adults worldwide had elevated TC levels in 2008 [4]. Moreover, the levels of both TC and non-HDL-C declined globally between 1980 and 2018, particularly in high-income North American countries, including the United States [4]. Despite this, from 2015 to 2018, 27.8% of US adults had raised LDL-C levels, 17.2% had low HDL-C levels, and 21.1% had elevated TG levels [5,6]. Therefore, as a major public health problem, interventions to promote the prevention and control of dyslipidemia are of great importance.

Dyslipidemia is co-caused by genetic and environmental factors [1]. Recently, studies have reported the harmful influence of environmental elements on lipid metabolism, such as heavy metals, air pollutants, polycyclic aromatic hydrocarbons, etc. [7,8,9]. Furthermore, exposure to environmental factors, notably heavy metals, may contribute to dyslipidemia through oxidative stress and inflammation [10,11,12]. Concentrations of blood heavy metals were considered more reliable indicators, reflecting the actual exposure levels of the body. Moreover, epidemiological studies have consistently revealed that the level of blood Pb was positively associated with the risk of dyslipidemia, but for other heavy metals, there was no definitive conclusion [10,13,14,15,16]. However, even low levels of exposure can pose chronic health risks, as people are prone to exposure to heavy metals in their daily lives through various ways, including contaminated air or soil, drinking water, or food [17,18,19]. Hence, avoiding heavy metal exposure in daily life is extremely difficult. Since dietary factors are closely associated with dyslipidemia [1], improving dyslipidemia caused by blood heavy metals exposure through dietary intervention may be a new approach.

Dietary fiber refers to non-digestible forms of carbohydrates, and numerous experimental studies have shown that dietary fiber is associated with the regulation of body weight and lipid levels [20,21]. Observational epidemiological studies in adults have also shown that dietary fiber is inversely related to body weight and contributes to modifying abnormal blood lipid levels [22,23]. In addition, increasing dietary fiber intake is the first-line therapy for patients with dyslipidemia who may receive an effective improvement [5]. Regretfully, most US adults did not meet the recommendation of dietary fiber intake, and the average intake of dietary fiber for adults globally continues to be low (<20 g/day) [24,25]. For this, there is an urgent need to promote increased dietary fiber intake among US adults, which may not only improve the dyslipidemia caused by heavy metal exposure but also bring many other health benefits [24].

In conclusion, blood Pb and dietary fiber may independently and inversely affect blood lipid levels. However, it is still unclear whether they have interactive effects in regulating dyslipidemia. Therefore, we used the data from the 2011–2016 cycles of the NHANES database to examine the association between blood Pb levels and dyslipidemia risk among US adults and whether dietary fiber can relieve the risk of dyslipidemia caused by Pb exposure. It is difficult to avoid exposure to Pb in people’s daily lives. Therefore, it is crucial to explore alternative strategies that are both practical and efficient in mitigating the harmful impact of lead on blood lipids. The purpose of this study was to provide a scientific foundation for preventing dyslipidemia and promoting lipid health through dietary fiber under exposure to Pb.

## 2. Material and Methods

### 2.1. Study Population

**Figure 1:**
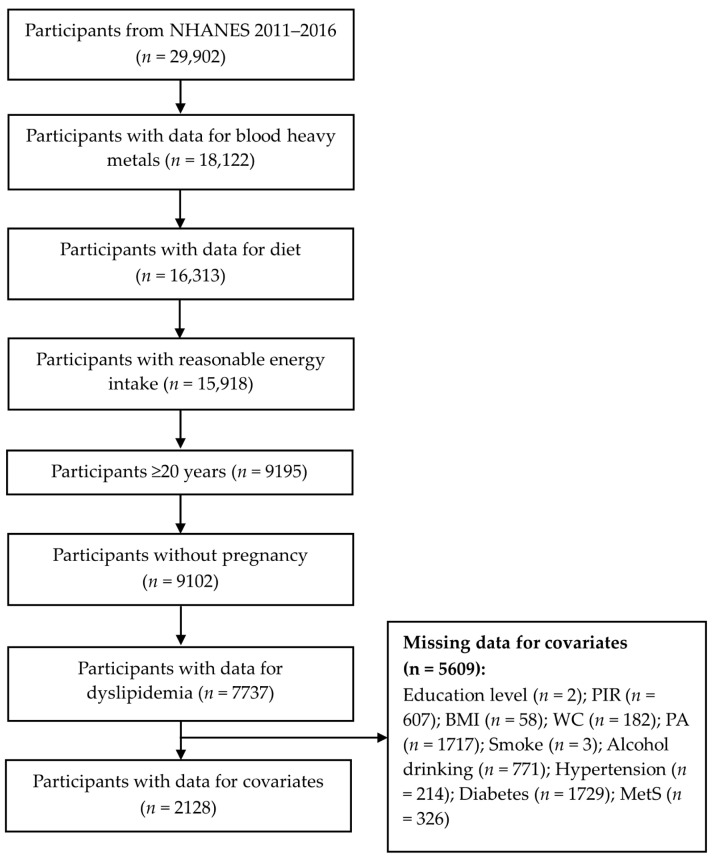
Flow chart of participants screening in this study. PIR, ratio of family income to poverty; WC, waist circumference; BMI, body mass index; PA, physical activity; MetS, metabolic syndrome.

Publicly accessible data from the NHANES (a nationwide cross-sectional survey) 2011–2016 cycles were used and are available at https://www.cdc.gov/nchs/nhanes/ (accessed on 11 August 2023). According to the screening criteria, we excluded those without data for blood heavy metals and diet and those with unreasonable energy intake (<600 kcal/day or >5000 kcal/day), age < 20 years, current pregnancy, and missing data on dyslipidemia diagnosis and selected covariates. Finally, we included 2128 participants for the subsequent analysis (Figure 1).

### 2.2. Measurements of Heavy Metals in Blood by ICP-MS

Inductively coupled plasma mass spectrometry (ICP-MS) was conducted to determine heavy metal concentrations (Pb, lead; Cd, cadmium; Mn, manganese; THg, total mercury) in whole blood samples. Detailed information about the NHANES laboratory processes has been described in previous studies [26,27]. All reported test results also conform to appropriate quality control and quality assurance standards [26,28]. The lower limit of detection (LLOD) for heavy metals was as follows: 2011–2012 cycle: Pb, 0.25 µg/dL; Cd, 0.16 µg/L; Mn, 1.06 µg/L; THg, 0.16 µg/L; and 2013–2016 cycles: Pb, 0.07 µg/dL; Cd, 0.10 µg/L; Mn, 0.99 µg/L; and THg, 0.28 µg/L. As for the results below the lower limit of detection, the specific value (LLOD/√2), was used to fill in missing values.

### 2.3. Definition of Dyslipidemia

Dyslipidemia was defined as the presence of one of the conditions below in the participants: (1) self-reported dyslipidemia, (2) currently using hypolipidemic drugs, (3) total cholesterol (TC) ≥ 200 mg/dL, (4) TG ≥ 150 mg/dL, (5) LDL-C ≥ 130 mg/dL, and (6) HDL-C < 50 mg/dL (female) or <40 mg/dL (male) [29].

### 2.4. Dietary Assessment

Dietary information from participants was collected through 24 h recall interviews conducted on two non-consecutive days. The first day’s data were collected face to face at the Mobile Examination Center (MEC), while the second day’s data were obtained via telephone 3 to 10 days later. The food items and drinks were translated into grams. Nutrient values were calculated through the US Department of Agriculture Nutrient Database and the Food Patterns Equivalents Database [29].

According to the previous study, the first day’s data were used following the National Cancer Institute’s analytic recommendations because it provided a reliable estimate of mean intake at the population level [30]. Furthermore, we utilized only the first day’s data rather than the average of the two days, thus preventing a loss of study power and sample size and avoiding the use of dietary data obtained in two different ways. An energy density approach was used, which calculated energy-adjusted total dietary fiber intake (g/1000 kcal/day). Based on the US Dietary Guidelines 2020–2025, a daily dietary fiber intake of 14 g/1000 kcal/day was considered an adequate intake [31].

### 2.5. Covariates Definition

Based on the previous studies, the following factors were chosen as potential confounders in this study [7,32,33]: First, demographic data, including age (continuous variable), gender (male, female), education level (below high school, equivalent to high school, or college or above), race (Mexican American, non-Hispanic White, non-Hispanic Black, or other races), PIR (<1.3, low-income; 1.3–3.5, middle-income; >3.5, high-income) [29], and marital status (never married, married/cohabiting, widowed/divorced/separated) [34].

Second, lifestyles, including smoke, alcohol drinking, and physical activity (continuous variable). Smoke was grouped as never smokers (smoked less than 100 cigarettes in their whole life), current smokers (smoked at least 100 cigarettes in their whole life, still smoking now), and former smokers (smoked at least 100 cigarettes in their whole life, quit smoking now) [35]. Alcohol drinking was grouped as non-drinkers (<12 drinks/year), light-to-moderate drinkers (female, <1 drink/day; male, <2 drinks/day), and heavy drinkers (female, ≥1 drink/day; male, ≥2 drinks/day) [35]. Metabolic equivalent (MET) values differ depending on the type of physical activity, and we estimated the value of physical activity (MET·hour/week) using the recommended MET values from the NHANES [32].

Finally, other selected covariates, including BMI (continuous variable), waist circumference (continuous variable), diabetes, hypertension, and MetS. Participants were defined as having diabetes by the presence of one of the following conditions: (1) self-reported diabetes, (2) currently using hypoglycemic drugs, (3) fasting plasma glucose ≥ 7.0 mmol/L, (4) OGTT 2 h plasma glucose ≥ 11.1 mmol/L, or (5) glycated hemoglobin A 1c ≥ 6.5% [29]. Participants were defined as having hypertension if they had an average of three systolic/diastolic blood pressure (SBP/DBP) measurements ≥140/90 mmHg, self-reported hypertension, or were currently using antihypertensive drugs [29]. Participants were defined as having MetS by the presence of three or more of the following conditions: (1) WC ≥ 102 cm (male) or ≥88 cm (female); (2) TG ≥ 150 mg/dL or currently using drugs to reduce TG levels; (3) HDL-C < 50 mg/dL (female) or <40 mg/dL (male), or currently using drugs to increase HDL-C levels; (4) SBP/DBP ≥ 130/85 mmHg or currently using antihypertensive drugs; (5) fasting plasma glucose ≥ 5.56 mmol/L or currently using hypoglycemic drugs [36].

### 2.6. Statistical Analyses

All statistical analyses considered the complex survey design of NHANES to ensure national representation. According to NHANES official recommendations, a rule of “the least common denominator” was applied to choose the appropriate sample weight, and the six-year sample weight was constructed for the subsequent analysis.

Weighted mean ± standard error (SE) or numbers (weighted proportions) were utilized to characterize continuous and categorical data, respectively. Differences between the two groups (dyslipidemia and non-dyslipidemia) were compared using the weighted *t*-test (continuous variables) and the weighted chi-squared test (categorical variables).

A weighted logistic regression was used to calculate odds ratios (ORs) and 95% confidence intervals (CIs). The crude model was unadjusted. Model 1 was adjusted for age (continuous), gender, race, education level, PIR, marital status, BMI (continuous), and PA (continuous). Model 2 was fully adjusted for potential confounders, which further adjusted for WC (continuous), smoke, alcohol drinking, MetS, hypertension, and diabetes based on Model 1. In addition, the multi-metal model was constructed to adjust other heavy metals based on Model 2. The dose–response relationship between blood heavy metals and dyslipidemia risk was explored using a weighted restricted cubic spline (RCS) analysis. The reference value was set at the 50th percentile, and the knots (*n* = 4) were set at the 5th, 35th, 65th, and 95th percentiles.

The interactive effect of blood Pb and dietary fiber was tested by constructing a cross-product term of blood Pb levels (continuous variable) and total dietary fiber intake (binary variable, <14 or ≥14 g/1000 kcal/day) in the fully adjusted model. In addition, the interactive effect of blood Pb and other variables was also tested through a similar method. We also performed subgroup analyses to test the robustness of the results and to identify high-risk populations. All statistical analyses were conducted using R (Version, 4.1.2). A two-tailed *p*-value < 0.05 was deemed to be statistically significant.

## 3. Results

### 3.1. Baseline Characteristics

As shown in Table 1, among the 2128 US adults, 1134 (51.9%) were males, and the weighted mean (SE) of age was 44.9 (0.6) years old. The participants with dyslipidemia tended to be older, non-Hispanic White, widowed/divorced/separated, and had a higher BMI, a lower physical activity level, a higher WC, and a higher prevalence of hypertension, diabetes, and MetS. Moreover, the participants with dyslipidemia also had a higher level of blood Pb.

### 3.2. The Association between Blood Heavy Metals and Dyslipidemia

As shown in Table 2, the level of blood Pb was positively associated with dyslipidemia risk in the crude model (OR = 1.44, 95% CI: 1.24–1.68). After adjusting for age (continuous), gender, race, education level, PIR, marital status, BMI (continuous), and PA (continuous) in Model 1, a significant association between blood Pb levels and dyslipidemia persisted (OR = 1.21, 95% CI: 1.04–1.40). Then, in Model 2, the association between blood Pb levels and dyslipidemia was still robust after fully adjusting for potential confounding factors (OR = 1.20, 95% CI: 1.03–1.40). Nevertheless, we did not find a significant association between the other three heavy metals and the risk of dyslipidemia.

The multi-metal model was constructed for a sensitive analysis, and the concentration of blood Pb was also positively associated with dyslipidemia after fully adjusting for covariates and other heavy metals (OR = 1.20, 95% CI: 1.03–1.40).

**Figure 2:**
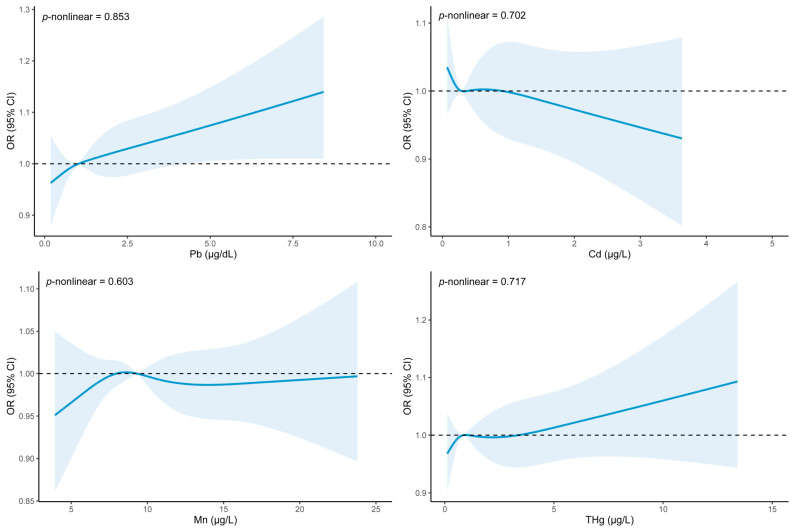
The association between dyslipidemia and blood heavy metals by weighted RCS analysis, NHANES 2011–2016. Adjusted for age (continuous), gender, race, education level, PIR, marital status, BMI (continuous), PA (continuous), WC (continuous), smoke, alcohol drinking, MetS, hypertension, and diabetes.

Figure 2 demonstrates the dose–response relationship between blood heavy metals and dyslipidemia risk from the weighted RCS analysis. After fully adjusting for potential confounders, we found that when the level of blood Pb was higher than 4.24 µg/dL, the risk of dyslipidemia increased with the increased level of blood Pb. However, no association was found between other heavy metals and dyslipidemia.

### 3.3. Regulation of Dietary Fiber on Dyslipidemia Caused by Blood Pb Exposure

As shown in Supplementary Table S1, taking the inadequate intake of dietary fiber group (<14 g/1000 kcal/day) as a reference, we found a null association between dietary fiber and dyslipidemia in the fully adjusted model (OR = 1.05, 95% CI: 0.64–1.71). However, blood Pb and dietary fiber interacted with the risk of dyslipidemia (Supplementary Table S2), and the risk of dyslipidemia increased with the level of blood Pb but decreased with dietary fiber (OR = 0.68, 95% CI: 0.46–1.00, *p* = 0.049).

As shown in Table 3, after fully adjusting for potential confounding factors, a stronger association between dyslipidemia and blood Pb levels was revealed in the group with an inadequate intake of dietary fiber (OR = 1.26, 95% CI: 1.05–1.52). There was no significant association between blood Pb levels and dyslipidemia in the adequate intake of dietary fiber group (OR = 1.01, 95% CI: 0.72–1.42).

**Figure 3:**
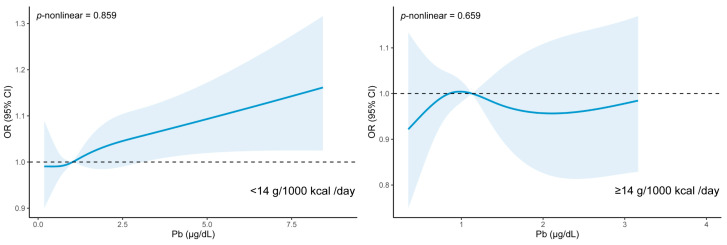
The association between dyslipidemia and the level blood Pb by weighted RCS analysis in the groups with or without adequate dietary fiber intake (g/1000 kcal/day), NHANES 2011–2016. Adjusted for age (continuous), gender, race, education level, PIR, marital status, BMI (continuous), PA (continuous), WC (continuous), smoke, alcohol drinking, MetS, hypertension, and diabetes.

From the weighted RCS analysis, Figure 3 presents the dose–response relationship between the level of blood Pb and dyslipidemia risk in the groups with or without an adequate dietary fiber intake. After fully adjusting for confounding factors, the association between blood Pb levels and the risk of dyslipidemia disappeared in the participants with an adequate intake of dietary fiber. Among those with an inadequate intake of dietary fiber, we discovered that when the level of blood Pb was only higher than 3.08 µg/dL, the risk of dyslipidemia increased with the increased level of blood Pb.

### 3.4. Subgroup Analysis

**Figure 4:**
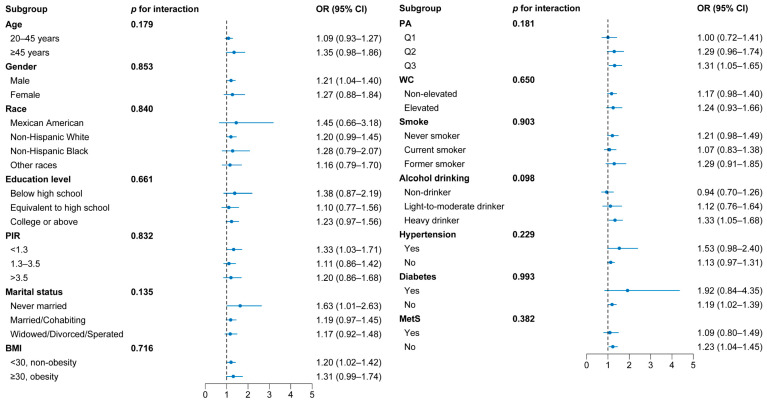
Forest plot of subgroup analysis of the association between dyslipidemia and blood Pb levels, NHANES 2011–2016.

Figure 4 shows the results of the association between dyslipidemia and blood Pb levels in different subgroups. After adjusting for other confounding factors, participants (males, low-income level, never married, non-obesity, high level of physical activity, heavy drinkers, no history of diabetes, and MetS) were more likely to suffer from dyslipidemia under Pb exposure, but their interactions with blood Pb were not statistically significant.

## 4. Discussion

Using data from the NHANES 2011–2016 cycles, a positive association was revealed between blood Pb levels and dyslipidemia. Moreover, a stronger association between dyslipidemia and blood Pb concentrations was detected in the group with inadequate dietary fiber intake, but a null association was observed in those with an adequate dietary fiber intake. This finding may provide a new way to alleviate the harmful impact of heavy metals on blood lipid health.

Recently, epidemiological studies have consistently discovered the association between blood Pb exposure and dyslipidemia. After adjusting for age, sex, BMI, family income, smoking, alcohol drinking, and physical activity, Kim et al. found that the level of blood Pb was positively associated with dyslipidemia risk among Korean adults [10]. Similarly, after adjusting for confounders, Wan et al. found a positive association between blood Pb levels and the risk of dyslipidemia in Chinese adults [14]. Furthermore, another two studies also found a consistent positive correlation between blood Pb levels and dyslipidemia in their multivariate adjusted models [13,15]. Based on the data from the NHANES 2011–2016 cycles, we discovered a positive association between dyslipidemia and blood Pb levels in this study. Currently, lead exposure may primarily contribute to lipid metabolism disorders through oxidative stress (OS) and inflammation [12,37,38,39]. Specifically, lead induces OS by damaging the antioxidant system and producing excessive reactive oxygen species (ROS) [37]. In addition, an overproduction of ROS can also trigger inflammation by activating the NF-kappaB pathway, which in turn exacerbates OS [39,40,41]. Ultimately, OS and inflammation result in impaired lipid metabolism and an elevated risk of dyslipidemia [12,37,38].

Except for heavy metals, dietary factors are also important for blood lipid health. Studies have shown that increased dietary fiber intake promotes the production of short-chain fatty acids (SCFAs) through gut microbial fermentation to improve lipid parameters in individuals [42,43]. Moreover, SCFAs exert antioxidant stress functions by regulating oxidoreductase, enhancing nuclear factor erythroid 2-related factor 2 (Nrf2), and restraining ROS and reactive nitrogen species (RNS) [44]. SCFAs also act as an anti-inflammatory agent by modulating cytokine generation and the function of immune cells [44]. In combination with the interactive effect analysis, we found that blood Pb and dietary fiber interacted with the risk of dyslipidemia. Since Pb promotes the occurrence of dyslipidemia through oxidative stress and inflammation, the beneficial impact of dietary fiber may explain why it could alleviate dyslipidemia caused by blood Pb exposure.

The subgroup analysis showed that there were differences in the association between blood Pb levels and the risk of dyslipidemia in populations with different characteristics. Consistent with the previous study, a significant association between blood Pb levels and dyslipidemia was found in the males rather than in the females, which may be due to males being more prone to exposure to Pb-contaminated environments, resulting in higher blood Pb levels [14]. Moreover, there were variations in racial composition among the dyslipidemia patients compared to the control group, which may be related to disparities in obesity, body fat percentage, and lean mass in different racial populations [45]. However, no significant differences were observed in the association between blood Pb levels and the risk of dyslipidemia among various racial populations. We also found that people who were unmarried, non-obese, and heavy drinkers and had a low income, a high physical activity level, and no history of diabetes or MetS were more likely to suffer from dyslipidemia under Pb exposure. Although their interactions with blood Pb were not statistically significant, the prevention of dyslipidemia in these populations also needs to be focused on in the future.

Our study also had some limitations. First, the cross-sectional design of this study makes it difficult to infer causality in comparison with prospective studies. Second, the assessment of dietary fiber intake relied on 24 h recall interviews, which may not reflect long-term intake. Third, due to the lack of data on Pb exposure levels in the external environment, it is impossible to analyze the impact of external exposure to Pb on blood lipid health.

## 5. Conclusions

There was a positive association between blood Pb levels and the risk of dyslipidemia among US adults. However, this association disappeared in those with an adequate intake of dietary fiber (≥14 g/1000 kcal/day). Therefore, increasing dietary fiber intake in the future might be a promising way to relieve dyslipidemia caused by blood Pb exposure, particularly in populations or regions with higher exposure levels of Pb or inadequate dietary fiber intake.

## Figures and Tables

**Table 1:** Baseline characteristics were described based on the two groups (dyslipidemia and non-dyslipidemia), NHANES 2011–2016.

Baseline Characteristics	Total (*n* = 2128)	Dyslipidemia (*n* = 1499)	Non-Dyslipidemia (*n* = 629)	*p*
Age, years	44.9 (0.6)	48.3 (0.6)	37.5 (0.7)	<0.001
Gender, *n* (%)				0.238
Male	1134 (51.9)	796 (50.9)	338 (54.1)	
Female	994 (48.1)	703 (49.1)	291 (45.9)	
Race, *n* (%)				0.034
Mexican American	249 (7.2)	179 (7.1)	70 (7.3)	
Non-Hispanic White	917 (69.4)	652 (70.8)	265 (66.4)	
Non-Hispanic Black	436 (11.1)	294 (9.9)	142 (13.7)	
Other races	526 (12.3)	374 (12.2)	152 (12.6)	
Education level, *n* (%)				0.278
Below high school	332 (11.2)	250 (11.9)	82 (9.6)	
Equivalent to high school	450 (18.8)	325 (19.0)	125 (18.3)	
College or above	1346 (70.0)	924 (69.0)	422 (72.0)	
PIR, *n* (%)				0.079
<1.3	615 (19.4)	419 (17.8)	196 (22.8)	
1.3–3.5	791 (37.1)	564 (37.7)	227 (35.6)	
>3.5	722 (43.6)	516 (44.5)	206 (41.6)	
Marital status, *n* (%)				<0.001
Never married	474 (21.6)	257 (16.8)	217 (32.3)	
Married/Cohabiting	1280 (63.5)	946 (65.9)	334 (58.1)	
Widowed/Divorced/Separated	374 (14.9)	296 (17.3)	78 (9.6)	
BMI, kg/m^2^	28.7 (0.2)	29.7 (0.2)	26.3 (0.3)	<0.001
PA, MET·hour/week	79.2 (3.9)	73.4 (4.4)	92.3 (6.5)	0.010
WC, centimeter	98.5 (0.6)	101.6 (0.6)	91.6 (0.7)	<0.001
Smoke, *n* (%)				0.523
Never smoker	1240 (58.9)	850 (58.0)	390 (61.0)	
Current smoker	412 (18.1)	296 (18.2)	116 (17.8)	
Former smoker	476 (23.0)	353 (23.8)	123 (21.2)	
Alcohol drinking, *n* (%)				0.341
Non-drinker	548 (20.1)	399 (21.1)	149 (17.9)	
Light-to-moderate drinker	230 (10.8)	163 (11.0)	67 (10.4)	
Heavy drinker	1350 (69.1)	937 (67.9)	413 (71.8)	
Prevalent hypertension, *n* (%)	789 (33.5)	653 (39.9)	136 (19.1)	<0.001
Prevalent diabetes, *n* (%)	363 (11.9)	317 (15.5)	46 (3.8)	<0.001
Prevalent MetS, *n* (%)	809 (35.1)	755 (47.5)	54 (7.5)	<0.001
Total dietary fiber intake, g/1000 kcal/day, *n* (%)				0.946
Inadequate intake, <14	1895 (89.9)	1335 (89.8)	560 (89.9)	
Adequate intake, ≥14	233 (10.1)	164 (10.2)	69 (10.1)	
Pb, µg/dL	1.26 (0.05)	1.36 (0.07)	1.04 (0.03)	<0.001
Cd, µg/L	0.43 (0.01)	0.43 (0.02)	0.43 (0.03)	0.959
Mn, µg/L	9.80 (0.09)	9.80 (0.11)	9.86 (0.16)	0.645
THg, µg/L	1.41 (0.09)	1.44 (0.10)	1.36 (0.11)	0.397

PIR, ratio of family income to poverty; WC, waist circumference; BMI, body mass index; PA, physical activity; MET, metabolic equivalent; MetS, metabolic syndrome. Weighted mean ± standard error (SE) or numbers (weighted proportions) were utilized to characterize continuous and categorical data, respectively. Differences between the two groups (dyslipidemia and non-dyslipidemia) were compared using the weighted *t*-test (continuous variables) and the weighted chi-squared test (categorical variables).

**Table 2:** The association between dyslipidemia and blood heavy metals by weighted logistic regression, NHANES 2011–2016.

Heavy Metals	Crude	*p*	Model 1	*p*	Model 2	*p*
Pb (µg/dL)	1.44 (1.24–1.68)	<0.001	1.21 (1.04–1.40)	0.014	1.20 (1.03–1.40)	0.020
Cd (µg/L)	1.01 (0.79–1.28)	0.959	1.07 (0.82–1.39)	0.601	0.87 (0.64–1.18)	0.356
Mn (µg/L)	0.99 (0.96–1.02)	0.639	0.99 (0.96–1.03)	0.689	1.00 (0.96–1.04)	0.991
THg (µg/L)	1.02 (0.97–1.08)	0.424	1.01 (0.96–1.07)	0.629	1.04 (0.97–1.10)	0.271

Crude, unadjusted. Model 1, adjusted for age (continuous), gender, race, education level, PIR, marital status, BMI (continuous) and PA (continuous). Model 2, further adjusted for WC (continuous), smoke, alcohol drinking, MetS, hypertension, and diabetes based on Model 1.

**Table 3:** The association between dyslipidemia and the level blood Pb by weighted logistic regression in the groups with or without adequate dietary fiber intake (g/1000 kcal/day), NHANES 2011–2016.

Total Dietary Fiber Intake	Case/Participants	%	OR (95% CI)	*p*
Inadequate intake, <14	1335/1895	70.4	1.26 (1.05–1.52)	0.015
Adequate intake, ≥14	164/233	70.4	1.01 (0.72–1.42)	0.946

Adjusted for age (continuous), gender, race, education level, PIR, marital status, BMI (continuous), PA (continuous), WC (continuous), smoke, alcohol drinking, MetS, hypertension, and diabetes.

## Data Availability

Data will be made available on request.

## Supplementary Materials

The following supporting information can be downloaded at https://www.mdpi.com/article/10.3390/nu15204434/s1: Table S1: The odds ratio of dyslipidemia with dietary fiber intake (<14 (reference) or ≥14 g/1000 kcal/day) by weighted logistic regression; Table S2: Multiplicative interaction between blood Pb levels and dietary fiber intake.

## References

[1] Kopin L., Lowenstein C. (2017). Dyslipidemia. Ann. Intern. Med..

[2] Berberich A.J., Hegele R.A. (2022). A Modern Approach to Dyslipidemia. Endocr. Rev..

[3] Hager M.R., Narla A.D., Tannock L.R. (2017). Dyslipidemia in patients with chronic kidney disease. Rev. Endocr. Metab. Disord..

[4] Pirillo A., Casula M., Olmastroni E., Norata G.D., Catapano A.L. (2021). Global epidemiology of dyslipidaemias. Nat. Rev. Cardiol..

[5] Agarwala A., Petersen K.S., Jafari F., Kris-Etherton P.M. (2022). Dietary management of dyslipidemia and the impact of dietary patterns on lipid disorders. Prog. Cardiovasc. Dis..

[6] Tsao C.W., Aday A.W., Almarzooq Z.I., Alonso A., Beaton A.Z., Bittencourt M.S., Boehme A.K., Buxton A.E., Carson A.P., Commodore-Mensah Y. (2022). Heart Disease and Stroke Statistics-2022 Update: A Report From the American Heart Association. Circulation.

[7] Cakmak S., Mitchell K., Lukina A., Dales R. (2023). Do blood metals influence lipid profiles? Findings of a cross-sectional population-based survey. Environ. Res..

[8] Hu M., Wei J., Hu Y., Guo X., Li Z., Liu Y., Li S., Xue Y., Li Y., Liu M. (2023). Long-term effect of submicronic particulate matter (PM(1)) and intermodal particulate matter (PM(1-2.5)) on incident dyslipidemia in China: A nationwide 5-year cohort study. Environ. Res..

[9] Wang Q., Xu X., Zeng Z., Hylkema M.N., Cai Z., Huo X. (2020). PAH exposure is associated with enhanced risk for pediatric dyslipidemia through serum SOD reduction. Environ. Int..

[10] Kim D.W., Ock J., Moon K.W., Park C.H. (2022). Association between Heavy Metal Exposure and Dyslipidemia among Korean Adults: From the Korean National Environmental Health Survey, 2015–2017. Int. J. Environ. Res. Public Health.

[11] Werder E.J., Beier J.I., Sandler D.P., Falkner K.C., Gripshover T., Wahlang B., Engel L.S., Cave M.C. (2020). Blood BTEXS and heavy metal levels are associated with liver injury and systemic inflammation in Gulf states residents. Food Chem. Toxicol..

[12] Esteve E., Ricart W., Fernandez-Real J.M. (2005). Dyslipidemia and inflammation: An evolutionary conserved mechanism. Clin. Nutr..

[13] Xu H., Mao Y., Xu B., Hu Y. (2021). Low-level environmental lead and cadmium exposures and dyslipidemia in adults: Findings from the NHANES 2005–2016. J. Trace Elem. Med. Biol..

[14] Wan H., Wang D., Liang Y., He Y., Ma Q., Li T., He Y., Guo H., Wang J., Li Z. (2023). Single and combined associations of blood lead and essential metals with serum lipid profiles in community-dwelling adults. Front. Nutr..

[15] Zhu X., Fan Y., Sheng J., Gu L., Tao Q., Huang R., Liu K., Yang L., Chen G., Cao H. (2021). Association Between Blood Heavy Metal Concentrations and Dyslipidemia in the Elderly. Biol. Trace Elem. Res..

[16] Cho H.W., Kim S.H., Park M.J. (2020). An association of blood mercury levels and hypercholesterolemia among Korean adolescents. Sci. Total Environ..

[17] Duan W., Xu C., Liu Q., Xu J., Weng Z., Zhang X., Basnet T.B., Dahal M., Gu A. (2020). Levels of a mixture of heavy metals in blood and urine and all-cause, cardiovascular disease and cancer mortality: A population-based cohort study. Environ. Pollut..

[18] Cao H.M., Yang Y.Z., Huang B.Y., Zhang Y., Wu Y., Wan Z., Ma L. (2023). A cross-sectional study of the association between heavy metals and pan-cancers associated with sex hormones in NHANES 1999–2018. Environ. Sci. Pollut. Res. Int..

[19] Swayze S., Rotondi M., Kuk J.L. (2021). The Associations between Blood and Urinary Concentrations of Metal Metabolites, Obesity, Hypertension, Type 2 Diabetes, and Dyslipidemia among US Adults: NHANES 1999–2016. J. Environ. Public Health.

[20] Barber T.M., Kabisch S., Pfeiffer A.F.H., Weickert M.O. (2020). The Health Benefits of Dietary Fibre. Nutrients.

[21] Stephen A.M., Champ M.M., Cloran S.J., Fleith M., van Lieshout L., Mejborn H., Burley V.J. (2017). Dietary fibre in Europe: Current state of knowledge on definitions, sources, recommendations, intakes and relationships to health. Nutr. Res. Rev..

[22] Howarth N.C., Huang T.T., Roberts S.B., McCrory M.A. (2005). Dietary fiber and fat are associated with excess weight in young and middle-aged US adults. J. Am. Diet. Assoc..

[23] Shinozaki K., Okuda M., Sasaki S., Kunitsugu I., Shigeta M. (2015). Dietary Fiber Consumption Decreases the Risks of Overweight and Hypercholesterolemia in Japanese Children. Ann. Nutr. Metab..

[24] Gill S.K., Rossi M., Bajka B., Whelan K. (2021). Dietary fibre in gastrointestinal health and disease. Nat. Rev. Gastroenterol. Hepatol..

[25] Soliman G.A. (2019). Dietary Fiber, Atherosclerosis, and Cardiovascular Disease. Nutrients.

[26] Caudill S.P., Schleicher R.L., Pirkle J.L. (2008). Multi-rule quality control for the age-related eye disease study. Stat. Med..

[27] Tanner S.D., Baranov V.I., Bandura D.R. (2002). Reaction cells and collision cells for ICP-MS: A tutorial review. Spectrochim. Acta Part B Atom. Spectrosc..

[28] Westgard J.O., Barry P.L., Hunt M.R., Groth T. (1981). A multi-rule Shewhart chart for quality control in clinical chemistry. Clin. Chem..

[29] Liu Z.-Y., Wang C., Huang S.-Y., Lu X.-T., Yang Z.-J., Lan Q.-Y., Huang B.-X., Chen S., Li M.-C., Zhu H.-L. (2023). Does anti-inflammatory diet mitigate the deleterious effect of bisphenol A on mortality in US adults? Results from NHANES 2003–2016. Ecotoxicol. Environ. Saf..

[30] Recommendations on Potential Approaches to Dietary Assessent for Different Research Objectives Requiring Group-Level Estimates. https://dietassessmentprimer.cancer.gov/approach/table.html#intake.

[31] U.S. Department of Agriculture, U.S. Department of Health and Human Services (2020). Dietary Guidelines for Americans, 2020–2025.

[32] Tian X., Xue B., Wang B., Lei R., Shan X., Niu J., Luo B. (2022). Physical activity reduces the role of blood cadmium on depression: A cross-sectional analysis with NHANES data. Environ. Pollut..

[33] Planchart A., Green A., Hoyo C., Mattingly C.J. (2018). Heavy Metal Exposure and Metabolic Syndrome: Evidence from Human and Model System Studies. Curr. Environ. Health Rep..

[34] Guo X., Wu B., Hu W., Wang X., Su W., Meng J., Lowe S., Zhao D., Huang C., Liang M. (2023). Associations of perchlorate, nitrate, and thiocyanate with metabolic syndrome and its components among US adults: A cross-sectional study from NHANES. Sci. Total Environ..

[35] Bao W., Liu B., Rong S., Dai S.Y., Trasande L., Lehmler H.J. (2020). Association Between Bisphenol A Exposure and Risk of All-Cause and Cause-Specific Mortality in US Adults. JAMA Netw. Open.

[36] Alberti K.G., Eckel R.H., Grundy S.M., Zimmet P.Z., Cleeman J.I., Donato K.A., Fruchart J.C., James W.P., Loria C.M., Smith S.C. (2009). Harmonizing the metabolic syndrome: A joint interim statement of the International Diabetes Federation Task Force on Epidemiology and Prevention; National Heart, Lung, and Blood Institute; American Heart Association; World Heart Federation; International Atherosclerosis Society; and International Association for the Study of Obesity. Circulation.

[37] Lopes A.C., Peixe T.S., Mesas A.E., Paoliello M.M. (2016). Lead Exposure and Oxidative Stress: A Systematic Review. Rev. Environ. Contam. Toxicol..

[38] Jomova K., Valko M. (2011). Advances in metal-induced oxidative stress and human disease. Toxicology.

[39] Attafi I.M., Bakheet S.A., Ahmad S.F., Belali O.M., Alanazi F.E., Aljarboa S.A., Al-Alallah I.A., Korashy H.M. (2022). Lead Nitrate Induces Inflammation and Apoptosis in Rat Lungs Through the Activation of NF-kappaB and AhR Signaling Pathways. Environ. Sci. Pollut. Res. Int..

[40] Flohe L., Brigelius-Flohe´ R., Saliou C., Traber M.G., Packer L. (1997). Redox regulation of NF-kappa B activation. Free Radic. Biol. Med..

[41] Mittal M., Siddiqui M.R., Tran K., Reddy S.P., Malik A.B. (2014). Reactive oxygen species in inflammation and tissue injury. Antioxid. Redox Signal..

[42] Cronin P., Joyce S.A., O’Toole P.W., O’Connor E.M. (2021). Dietary Fibre Modulates the Gut Microbiota. Nutrients.

[43] Makki K., Deehan E.C., Walter J., Backhed F. (2018). The Impact of Dietary Fiber on Gut Microbiota in Host Health and Disease. Cell Host Microbe.

[44] Liu P., Wang Y., Yang G., Zhang Q., Meng L., Xin Y., Jiang X. (2021). The role of short-chain fatty acids in intestinal barrier function, inflammation, oxidative stress, and colonic carcinogenesis. Pharmacol. Res..

[45] Liu B., Du Y., Wu Y., Snetselaar L.G., Wallace R.B., Bao W. (2021). Trends in obesity and adiposity measures by race or ethnicity among adults in the United States 2011-18: Population based study. BMJ.

