# Supplementary material for: Adequate Intake of Dietary Fiber May Relieve the Detrimental Impact of Blood Lead on Dyslipidemia among US Adults: A Study of Data from the National Health and Nutrition Examination Survey Database
Source: Nutrients. 2023 Oct 19;15(20):4434. doi: 10.3390/nu15204434 (PMC10610417; doi:10.3390/nu15204434)
Supplement: Supplementary file 1 [file nutrients-15-04434-s001.zip › nutrients-2657261-SI.pdf]

**Supplementary Table S1.** The odds ratio of dyslipidemia with dietary fiber intake (<14 (reference) or ⩾14 g/1000 kcal/day) by weighted logistic regression.

| Model | β | OR (95%CI) | *p* |
|---|---|---|---|
| **Crude** | 0.011 | 1.011 (0.721-1.419) | 0.946 |
| **Model 1** | 0.018 | 1.018 (0.670-1.546) | 0.932 |
| **Model 2** | 0.047 | 1.049 (0.643-1.710) | 0.843 |

Crude, unadjusted. Model 1, adjusted for age (continuous variable), gender, race, education level, PIR, marital status, BMI (continuous variable) and PA (continuous variable). Model 2, further adjusted for WC (continuous variable), smoke, alcohol drinking, MetS, hypertension, and diabetes based on Model 1.

**Supplementary Table S2.** Multiplicative interaction between blood Pb levels and dietary fiber intake.

| Model | β | OR (95%CI) | *p* |
|---|---|---|---|
| Dietary fiber | 0.488 | 1.629 (0.752-3.529) | 0.205 |
| Pb | 0.234 | 1.264 (1.054-1.517) | **0.014** |
| Pb*Dietary fiber | −0.385 | 0.681 (0.464-0.998) | **0.049** |

Pb (continuous variable); dietary fiber (binary variable, <14 (reference) or ⩾14 g/1000 kcal/day). The two are multiplied into the fully adjusted weighted logistic regression model.

Adjusted for age (continuous variable), gender, race, education level, PIR, marital status, BMI (continuous variable), PA (continuous variable), WC (continuous variable), smoke, alcohol drinking, MetS, hypertension and diabetes.
